# Adrenoceptor Expression during Intervertebral Disc Degeneration

**DOI:** 10.3390/ijms21062085

**Published:** 2020-03-18

**Authors:** Johannes Kupka, Annika Kohler, Karima El Bagdadi, Richard Bostelmann, Marco Brenneis, Christoph Fleege, Danny Chan, Frank Zaucke, Andrea Meurer, Marcus Rickert, Zsuzsa Jenei-Lanzl

**Affiliations:** 1 Dr. Rolf M. Schwiete Research Unit for Osteoarthritis, Orthopedic University Hospital Friedrichsheim gGmbH, 60528 Frankfurt/Main, Germanyannika.kohler@friedrichsheim.de (A.K.); karima.elbagdadi@friedrichsheim.de (K.E.B.); marco.brenneis@friedrichsheim.de (M.B.); frank.zaucke@friedrichsheim.de (F.Z.); andrea.meurer@friedrichsheim.de (A.M.); marcus.rickert@friedrichsheim.de (M.R.); 2 Clinic of Neurosurgery, Heinrich Heine University, 40225 Duesseldorf, Germany; richard.bostelmann@med.uni-duesseldorf.de; 3 School of Biomedical Sciences, The University of Hong Kong, Pokfulam, Hong Kong, China; chand@hku.hk

**Keywords:** intervertebral disc (IVD), IVD degeneration (IVDD), sympathicus, adrenoceptors

## Abstract

Healthy and degenerating intervertebral discs (IVDs) are innervated by sympathetic nerves, however, adrenoceptor (AR) expression and functionality have never been investigated systematically. Therefore, AR gene expression was analyzed in both tissue and isolated cells from degenerated human IVDs. Furthermore, human IVD samples and spine sections of wildtype mice (WT) and of a mouse line that develops spontaneous IVD degeneration (IVDD, in SM/J mice) were stained for ARs and extracellular matrix (ECM) components. In IVD homogenates and cells *α1a-, α1b-, α2a-, α2b-, α2c-, β1-*, and *β2*-AR genes were expressed. In human sections, *β2*-AR was detectable, and its localization parallels with ECM alterations. Similarly, in IVDs of WT mice, only β2-AR was expressed, and in IVDs of SM/J mice, β2AR expression was stronger accompanied by increased collagen II, collagen XII, decorin as well as decreased cartilage oligomeric matrix protein expression. In addition, norepinephrine stimulation of isolated human IVD cells induced intracellular signaling via ERK1/2 and PKA. For the first time, the existence and functionality of ARs were demonstrated in IVD tissue samples, suggesting that the sympathicus might play a role in IVDD. Further studies will address relevant cellular mechanisms and thereby help to develop novel therapeutic options for IVDD.

## 1. Introduction

Degenerative changes of the intervertebral disc (IVD) represent a severe health issue resulting in devastating and disabling symptoms like pain, muscle weakness, numbness, and dysesthesia of the upper and lower limbs [1]. In particular, low back pain is the leading cause of years lived with disability in developed and developing countries [2]. Like articular cartilage tissue, the almost completely avascular and aneural IVD exhibits very limited or no regeneration capacity [3,4]. The standard treatment option of advanced IVD degeneration (IVDD) is surgical spinal fusion in the form of a spondylodesis with an increased frequency of 500% between 1990 and 2011 in the USA [5].

The IVD consists of two main parts with different embryologic origins: The ligamentous annulus fibrosus (AF) and the gelatinous nucleus pulposus (NP) [6]. It is well known that multiple changes in cellular processes, matrix composition, and functionality occur during the development of IVDD [3]. For example, a decreasing proteoglycan concentration leads to reduced hydration of the NP and lowers the mechanical resistance of the IVD [7]. Furthermore, in the AF, there is a shift from a fibrocytic to a chondrocytic phenotype accompanied by increased type II collagen expression [8]. There is evidence that IVDD pathogenesis is a multifactorial process influenced by age, gender, environmental, and hereditary factors [3,8,9,10]. Even though our knowledge of the mechanisms leading to disc destruction has increased over the past years, the detailed etiology and pathophysiology are not properly understood.

During the past decades, the role of peripheral sympathetic nerve fibers and their neurotransmitters have gained in importance with regard to degeneration and regeneration of joint tissues like articular cartilage [11]. The key enzyme of the sympathicus is tyrosine hydroxylase (TH), which controls the biosynthesis of the major catecholamines like, e.g., norepinephrine (NE) [12]. NE acts dose-dependently via different subtypes of alpha and beta-adrenoceptors (ARs, namely *α1a, α1b, α1d, α2a, α2b, α2c, β1, β*2, and *β3*), which results in activation of two major intracellular signaling pathways, ERK1/2 and PKA [11,13]. Studies on chondrocytes and chondrogenically differentiating mesenchymal stem cells demonstrated that sympathetic neurotransmitters, especially norepinephrine (NE), influence cartilage physiology and pathophysiology in a catabolic manner and mainly via *α2a-* and *β2*-AR [11]. Although there are many analogies between human intervertebral discs and articular joints [4], only few studies investigated sympathetic nerve fibers, sympathetic neurotransmitters, or their receptors in the context of IVDD. Recently, nerve fiber ingrowth into the IVD during degeneration was observed, however, these studies focused only on sensory nerves [14], on AR expression in dorsal root ganglia [15], or on pain mechanisms [14,16]. The only study on sympathetic nerve fibers in the IVD was performed recently by Barczewska et al., showing that sympathetic nerve fibers are present in lumbar IVDs with pathological changes [17]. At present, still no data exists investigating the expression of ARs in the IVD, which represents a prerequisite for sympathetic neurotransmitter action.

Therefore, the aim of this study was to characterize the expression profile of all AR subtypes in IVD tissue, as well as in isolated IVD cells, to specify their localization, and to correlate this expression profile with the grade of degeneration. In addition, the expression of TH, the-rate limiting enzyme of NE biosynthesis was examined.

## 2. Results

### 2.1. AR and TH Gene Expression in Human IVD Tissue Samples

**Figure 1:**
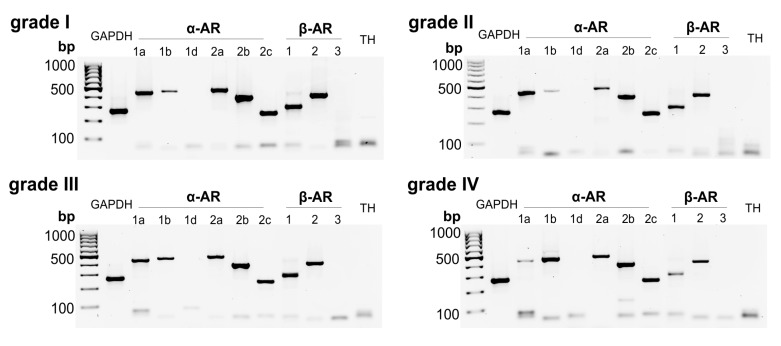
Adrenoceptor (AR) and tyrosine hydroxylase (TH) gene expression in human intervertebral disc (IVD) tissue analyzed by RT-PCR. Gene expression of all known AR subtypes as well as of TH in human IVD tissue samples with different degree of degeneration (representative pictures of human IVD samples; 4-grade classification scale: Non-/moderately (grade I), moderately (grade II), significantly (grade III), and massively degenerated (grade IV) (*n* = 43)).

In human IVD tissue homogenates, different AR subtypes were detected: The receptors *α1a-* (97.7% of samples), *α1b*- (46.5%), *α2a-* (90.7%), *α2b*- (97.7%), *α2c-* (97.7%), β1- (100%), and *β2-*AR (100%) were highly expressed (Figure 1, Table 1). In contrast, *α1D-*, *β3*-AR, and TH genes were not detected (Figure 1). No fundamental changes were visible, depending on the degree of degeneration. Only a slight decrease in the receptors *α1a-, α2b, β1-*, and *β2*-AR was observed, while the expression of *α1b*-AR slightly rose with increasing degree of degeneration (Figure 1).

### 2.2. Correlation between AR and TH Gene Expression and the Degree of IVD Degeneration

**Figure 2:**
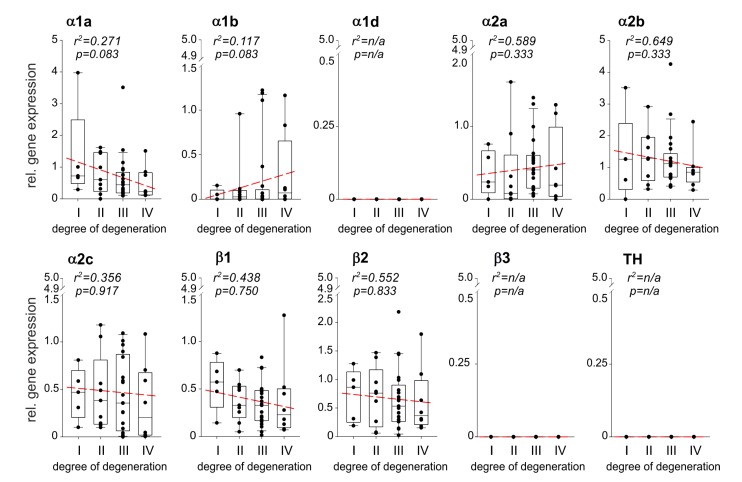
Correlation between AR and TH gene expression and the degree of IVD degeneration. Gene expression changes of the ARs as well as of the enzyme TH in different stages of IVD degeneration (gene expression level mean of stage I samples = 1 represented by the dashed-line). Each black circle represents an individual patient (*n* = 43). Data are presented as box plots, where the boxes represent the 25th to 75th percentiles, the lines within the boxes represent the median, and the lines outside the boxes represent the 10th and 90th percentiles. The red dotted lines represent the linear regressions for each gene analyzed.

Relative AR gene expression levels during IVDD were calculated and correlations were performed with the degree of degeneration (*n* = 43). Several AR genes were expressed at any stage of IVDD, the *α1a-, α1b-, α2a-, α2b-, α2c-, β1*-AR, and *β2*-AR (Figure 2). The expression of *α1a-, α2b-, α2c-, β1-*AR, and *β2-*AR increased by trend with the degree of degeneration, however, no significant correlation was observed (Figure 2). In contrast, *α1b-* and *α2a*-AR gene expression slightly but not significantly decreased with the degree of degeneration (Figure 2). The ARs *α1d* and *β3*, as well as TH, were not detected in any sample (Figure 2). No age- or gender-dependent differences were observed regarding AR or TH expression in human IVD samples.

### 2.3. Localization of ARs in Human Degenerated IVD Tissue

**Figure 3:**
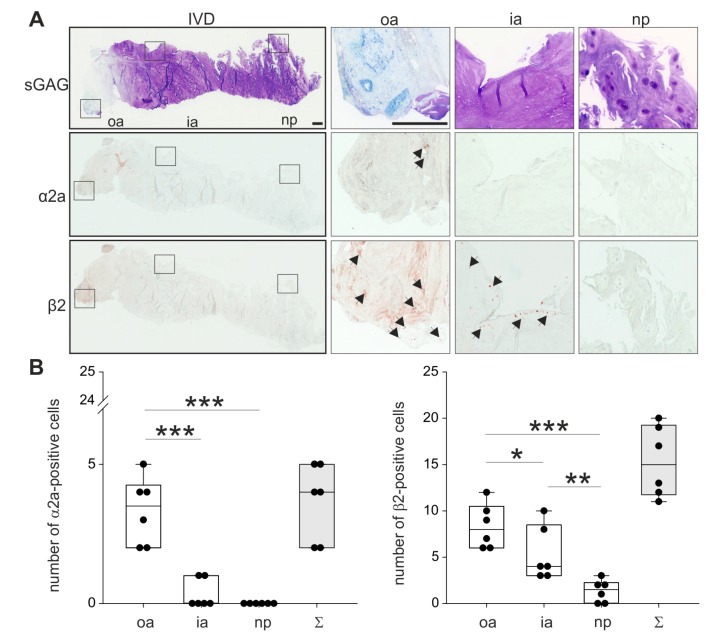
Localization and distribution of ARs. (**A**) Immunohistochemical analysis of the most prominent ARs (α2a-AR and β2-AR, black arrows) in human IVD sections (bars 500 μm). Squares in the left panel represent further magnified regions shown to the right (*n* = 3). (**B**) The number of α2a- and β2-positive cells in human IVD tissue (*n* = 3). Each black circle represents the number of α2a- or β2-positive cells per section of an individual patient (2 sections per patient analyzed). Data are presented as box plots, which are described in the legend to Figure 2. Significant *p*-values are presented as * *p* < 0.05, ** *p* < 0.01, or as *** *p* < 0.001.

In order to analyze the localization of the two most relevant ARs described in the literature, human IVD sections with degeneration grade II were immunostained for α2a- and β2-AR (no intact human IVD tissue was available with degeneration grades III or IV). Both ARs were detectable in the outer zone of AF, however, α2a-AR staining was not as intense as β2-AR (Figure 3; respective isotype stainings are presented in Supplementary Figure S1). Furthermore, β2-AR-positive cells were detected in the inner AF, while no α2a-AR staining was observed in this zone. In the NP, neither α2a-AR nor β2-AR was detectable (Figure 3). The quantification of α2a- and β2-ARs in 2 sections of 3 different donors confirmed this observation. In the outer annulus, significantly more cells were positive for α2a- (*p* = 0.04) and β2-AR (*p* < 0.001) compared to the inner annulus and also to the nucleus (α2a *p* < 0.001 and β2-AR *p* < 0.001). In addition, significantly more β2-AR-positive cells were counted in the inner annulus compared to the nucleus (*p* = 0.009). In contrast, no significant differences were detected between α2a-positive cells between inner annulus and nucleus (*p* = 0.459). We also performed a Western Blot to detect β2-AR, because this AR was expressed at the highest level. However, β2-AR could not be detected in human tissue samples (Supplementary Figure S5), possibly due to the low cell number and accordingly to the low cellular protein amount in the homogenates.

Abbreviations: sGAG—sulfated glycosaminoglycans, oa—outer annulus, ia—inner annulus, np—nucleus pulposus, Σ—sum of positive cells in oa + ia + np.

### 2.4. Associations of β2-AR Expression with Changes in ECM Expression in Human IVD Tissue

**Figure 4:**
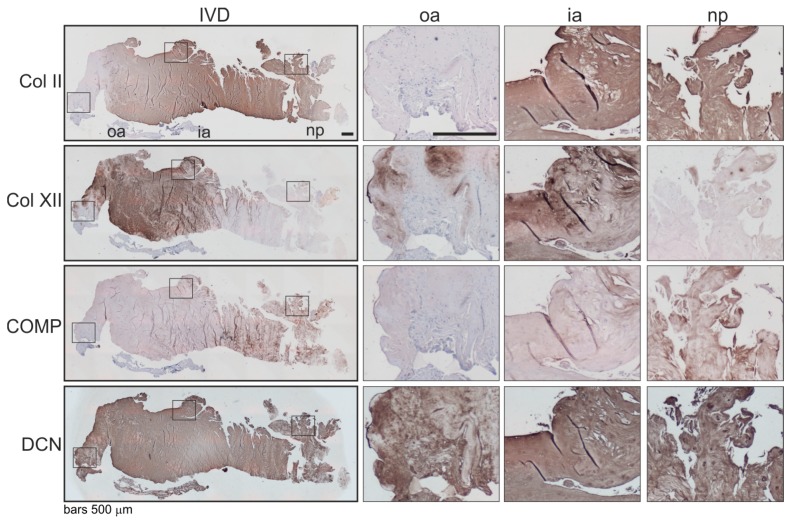
Localization and distribution of ECM proteins in human IVD tissue. Immunohistochemical analysis of type II collagen (Col II), type XII collagen (Col XII), cartilage oligomeric matrix protein (COMP) as well as decorin (DCN) in human IVD sections (bars 500 μm). Squares in the left panel represent further magnified regions shown to the right (*n* = 3). Abbreviations: oa—outer annulus, ia—inner annulus, np—nucleus pulposus, Σ—sum of positive cells in oa + ia + np.

Associations of the expression level and localization of the β2-AR with potential changes in the ECM expression were analyzed immunohistochemically. Since there were many analogies between human intervertebral discs and articular joints [4], we analyzed selected minor extracellular matrix proteins, which helped to mediate interactions between fibrils and other major matrix macromolecules and were, therefore, associated with articular cartilage degeneration [18] (type II —Col II, type XII collagen—Col XII, cartilage oligomeric matrix protein—COMP, decorin—DCN) [19,20,21]. In β2-AR-positive IVD areas, type II collagen and COMP staining was clearly reduced compared to IVD zones without a β2-AR signal (Figure 4). In contrast, strong type XII collagen and decorin staining were detected in β2-AR-positive IVD areas (Figure 4).

### 2.5. AR Protein Expression in Murine IVD Degeneration Models and Associations of ar Expression with Changes in ECM Expression in Murine IVD Tissue

**Figure 5:**
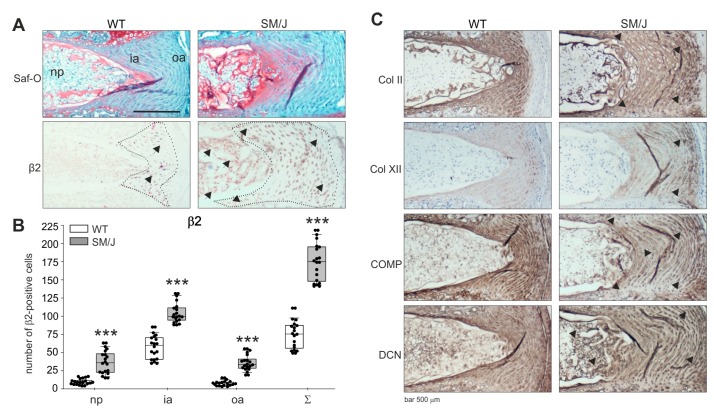
Localization of ARs and distribution of ECM proteins in murine IVD tissue. (**A**) Immunohistochemical analysis of β2-AR (black arrows and IVD regions indicated by the dotted line) and Col II, Col XII, COMP as well as DCN in IVD sections of WT and SM/J mice (bars 500 μm, *n* = 3). (**B**) The number of β2-positive cells in WT and SM/J IVD tissue (*n* = 3). Each black circle represents the number of β2-positive cells per section of an individual mouse (6 sections per mouse per genotype analyzed). Data are presented as box plots, which are described in the legend to Figure 2. Significant *p*-values against WT are presented as “***” *p* ≤ 0,0001. (**C**) Immunohistochemical analysis of type II collagen (Col II), type XII collagen (Col XII), cartilage oligomeric matrix protein (COMP) as well as decorin (DCN) in human IVD sections. Abbreviations: Saf-O—safranin-O staining, oa—outer annulus, ia—inner annulus, np—nucleus pulposus, Σ—sum of positive cells in oa + ia + np.

Since no healthy or highly degenerated human IVD sections were available, it was not possible to observe AR expression changes between healthy and degenerated stages [22]. In addition, it was not possible to draw conclusions from representative histological images of only one degeneration grade. Therefore, murine IVD sections of transgenic mice were analyzed in order to compare degenerated with healthy IVD sections regarding AR expression. Using safranin-O/fast green staining, the structure and grade of degeneration were demonstrated. IVD sections of SM/J mice showed clear signs of IVDD [23], while no degenerative changes were observed in WT disc tissue (WT: C57Bl/6, see Section 4.2.) (Figure 5). β2-AR was detectable in both WT and SM/J IVD sections. Similarly to the human IVD tissue, most β2-AR-positive cells were detected in the annulus fibrosus (Figure 5). In WT sections, this AR was predominantly expressed in the inner AF area. In contrast, β2-AR expression in SM/J samples spread into the degenerating outer AF and NP (Figure 5). α2a-AR was not detectable, neither in WT nor in SM/J samples (Supplementary Figure S2; respective isotype stainings for α2a-AR and β2-AR are presented in Supplementary Figure S3). The quantification of β2-ARs in 6 sections of 3 animals per genotype confirmed this observation. In the outer annulus, significantly more cells were positive for β2-AR in SM/J mice (*p* < 0.001) compared to WT mice and also in the inner annulus (*p* < 0.001) and in the nucleus (*p* < 0.001) (Figure 5).

Parallel to the β2-AR expression changes in the outer AF and NP, alterations in the expression of ECM components were observed in SM/J sections. Type II collagen staining intensity did not increase, but the type II collagen-positive chondrocytic area spread in the same manner as the β2-AR expression. Moreover, type XII collagen staining increased in the interterritorial ECM of SM/J IVDs, while COMP expression slightly decreased compared to WT samples. In addition, decorin staining became stronger in the pericellular and territorial ECM of SM/J mice (Figure 5).

### 2.6. AR and TH Gene Expression in Isolated Human IVD Cells and IVD Cell Response to NE

**Figure 6:**
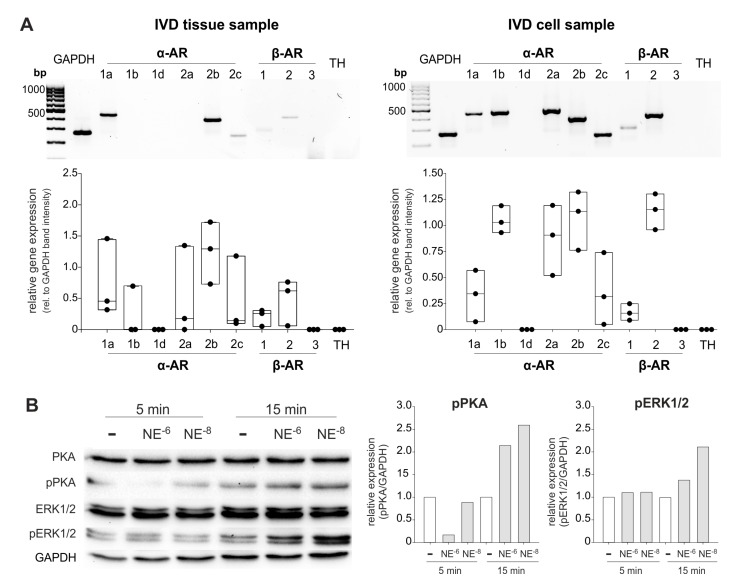
AR gene expression and NE-mediated activation of intracellular signaling pathways in human IVD cells. (**A**) Gene expression of different AR subtypes in untreated primary IVD cells isolated from three patients (representative RT-PCR image of one IVD donor) compared to the respective tissue sample. (**B**) Changes in PKA and ERK1/2 phosphorylation in isolated IVD cells after treatment with low or high NE concentrations (10^−8^ or 10^−6^ M) after 5 or 15 min ((*n* = 3); representative Western Blot images of cells isolated from one IVD donor). The expression of phosphorylated kinases (pPKA and pERK1/2) is shown relative to GAPDH expression (set as 1). White boxes indicate untreated controls, the grey boxes the NE-treatment groups.

In isolated IVD cells, a similar AR profile was detected at the mRNA level as in the IVD tissue (*α1a-, α2a-, α2b-, α2c-, β1-*, and *β2-*AR; Figure 6A). However, individual differences between the IVD cell samples were visible in contrast to IVD tissue. IVD cells of different donors always expressed the AR subtypes *α1a-, α2a-, α2b-, α2c-, β1-*, and *β2*-AR, but in cells of some patients *α1b-* or *β3-*AR was not detectable (Figure 5A, see also Supplementary Figure S4). Compared to the *GAPDH* expression, it seems that *β2-*AR expression was upregulated under culture conditions (Figure 6). We also performed *β2-*AR Western Blot and confirmed the expression of this AR in two IVD cell lysates (both with degeneration degree II, Supplementary Figure S5).

Treatment of isolated IVD cells with NE (10^−8^ or 10^−6^ M) for 5 or 15 min revealed that both the ERK1/2 and the PKA signaling pathway were activated. After 5 min, only a slight PKA phosphorylation was visible and only after treatment with 10^−8^ M NE. Here, ERK was not yet activated (Figure 6B). After 15 min, both NE concentrations induced both PKA and ERK1/2 phosphorylation, however, 10^−8^ M NE stimulation resulted in stronger ERK1/2 phosphorylation compared to the 10^−6^ M NE treated group, while no such concentration-dependent effect was observed regarding PKA activation (Figure 6B).

## 3. Discussion

Although the sympathetic innervation of healthy and degenerating IVDs is known for about 10 years [3], no study analyzing the expression of ARs during IVDD has been performed until now. Therefore, we investigated the expression of all AR subtypes as well as changes in AR expression in healthy and degenerated IVD tissue. Our study demonstrates that genes of almost all AR subtypes are expressed in human IVD tissue and can also be detected in isolated and expanded IVD cells. However, only the β2-AR was detected in human and murine sections in relevant amounts at the protein level. The β2-AR signal increased with the degree of degeneration and was accompanied by changes in the ECM expression. In addition, we demonstrated for the first time that human IVD cells are able to respond to NE by activating the PKA and ERK1/2 intracellular signaling pathways.

First, we analyzed human tissue homogenates prepared from IVDs with different degrees of degeneration and observed that apart from *α1D-* and *β3-*AR, all other AR subtypes were expressed at any stage of degeneration, suggesting that IVD tissue is able to respond to adrenergic stimulation at any time point. This would make sense given the fact that both healthy and degenerating IVD are innervated by sympathetic nerve fibers [17]. The fact that many AR subtypes were detected at the mRNA level is not surprising, because a similar AR expression pattern was described for human articular chondrocytes [24], which exhibit multiple phenotypic similarities to IVD cells [25].

However, AR expression at the mRNA level is not as decisive as the expression of functional AR proteins on the cell membrane. Therefore, we performed immunostainings and analyzed the expression and localization of the two most relevant ARs, the α2a- and β2-AR, in human and murine IVD sections. In human slightly degenerated IVD tissue, mainly β2-AR was detected and exclusively in the AF region. In contrast, only a weak α2a-AR signal was visible in the AF. Consistent with our observations in human disc tissue, only the β2-AR was detected in healthy murine IVDs and only in the AF area. Interestingly, the positive β2-AR signal spread out into the degenerating outer AF and also into the NP in highly degenerated murine IVDs. The absence of ARs in the NP area of healthy and slightly degenerated IVDs might be explained by the fact that only the AF is innervated by sympathetic nerves, consequently, neurotransmitters are released only to the AF region, which leads to an AR upregulation. However, exactly the opposite has been described in aorta muscle cells, hamster ovary cell line, or in HT29 cell line [26,27,28]. Another possible explanation could be the difference in the ECM structure between AF and NP and accordingly, the inhomogeneously distributed mechanical loading. While AF cells, surrounded by stiffer ligamentous ECM, are predominantly exposed to tensile strain, the NP cells are mainly under hydrostatic pressure due to their gelatinous matrix with high water content [29,30]. Recent studies described that ARs can function as sensors of cell membrane stretch in the vascular smooth muscle and mediate vasoconstriction [31,32]. Therefore, mechanical stimuli by overloading of the IVD might lead to β2-AR upregulation. This would explain the enhanced and expanded expression of β2-AR in highly degenerated IVDs of SM/J mice.

Concomitantly with increased and expanded β2-AR expression, changes in the ECM deposition were observed in degenerated murine IVD sections. The expanded type II collagen positive AF area is a clear sign of IVD degeneration. The cells there exhibit a more chondrocytic phenotype and produce enhanced amounts of type II collagen [17]. The increased type XII collagen expression in the AF might also indicate that resident cells try to respond to overloading and ongoing catabolic processes by enhanced matrix synthesis as an attempt to repair the tissue. The expression of type XII collagen has been shown to depend on age with decreasing amounts in older tissue [33]. Data from another study on hyaline cartilage suggest that type XII collagen might be necessary to provide a microenvironment that supports proper tissue formation [34]. With regard to function, collagen type XII in cartilage has been shown in areas with more organized collagen fibril orientation. Here, it might play a role in promoting alignment or in stabilizing such an organization, thereby creating a matrix capable of withstanding load-bearing forces [35]. The slightly decreased COMP expression and the increased pericellular and territorial decorin synthesis demonstrate degeneration-related catabolic changes as described earlier for articular chondrocytes [36,37]. COMP is a target of matrix metalloproteinases, including MMP-13 [38], which have been shown to be upregulated in chondrogenically differentiating mesenchymal stem cells as well as in cardiofibroblasts by targeting the β2-AR [18,39]. Thus, enhanced β2-AR expression due to mechanical overloading might contribute to the perpetuation of IVDD.

After analyzing AR expression in tissue samples, we investigated the AR expression profile in isolated human IVD cells. We found a very similar AR expression profile in isolated and expanded IVD cells as in IVD tissue homogenates indicating that cell culture conditions over more than 2 weeks do not substantially influence the AR expression. A similar phenomenon was observed in human articular chondrocytes after 7 days in monolayer culture [40]. However, *α1b-* and *β2-*AR expression was slightly upregulated under culture conditions. The reason for this observation might be that monolayer culture conditions lead to dedifferentiation processes, which in turn could result in AR regulation [40]. Indeed, also degeneration results in dedifferentiation, therefore, we believe that our monolayer culture conditions used in the present study and as performed by others [41] optimally mimic the phenotype and behavior of IVD cells in vivo. Furthermore, isolated IVD cells were able to respond to NE stimulation by PKA and ERK1/2 phosphorylation. A similar signaling response was shown for ARs in human articular chondrocytes as well as in synovial stem cells undergoing chondrogenic differentiation [42,43]. These findings provide the opportunity to perform experiments analyzing the functional response of isolated IVDs to sympathetic neurotransmitters, which is, therefore, the focus of our present and future work.

In summary, we demonstrated the existence of ARs, especially of β2AR, in IVD tissue for the first time. Its differential expression in healthy and degenerated IVD as well as the ability of isolated IVD cells to respond to NE stimulation suggests that the sympathicus might play a role in IVDD. Further studies will address its mechanistic contribution and thereby help to develop novel therapeutic options for IVDD.

## 4. Materials and Methods

### 4.1. Human IVD Tissue

IVD samples were obtained from patients undergoing spondylodesis at the University Hospitals of the Johann Wolfgang Goethe University Frankfurt and the Heinrich Heine University Düsseldorf. The samples were anonymized and according to the the Ethics Committees of both universities no approval was necessary. All experiments were performed in accordance with relevant guidelines and regulations. The experimental cohort included 43 patients (Table 2), at least 5 patients per group.

Samples were stored at −80 °C until analysis. The degree of degeneration was determined pre- and intraoperatively with a 4-grade classification scale according to the Modic and Pfirmann classification [44]: Non-/moderately (grade I), moderately (grade II), significantly (grade III), and massively degenerated (grade IV). In the majority of cases, the condition of the tissue made it impossible to distinguish between AF and NP, and therefore, their ratio was variable. In addition, few well-preserved IVD samples with AF and NP zones were taken for histological analysis. 

### 4.2. Murine IVD Samples

Murine IVDD models enabled the analysis of all degeneration stages, and especially the healthy situation could be investigated in contrast to human samples. The transgenic mice SM/J developed an early spontaneous disc degeneration [23]. As non-degenerated controls, spine sections of the respective wildtype animals were analyzed (C57BL/6 for SM/J mice;). IVD sections of C57BL/6, and SM/J, mice were kindly provided by Prof. Danny Chan (School of Biomedical Sciences, The University of Hong Kong, Hong Kong, China). Spine dissection, fixation, decalcification, and paraffin embedding was performed as described previously [23].

### 4.3. Adrenoceptor Gene Expression Analysis

The expression of the genes for all adrenoceptor subtypes (*α1a, α1b, α1d, α2a, α2b, α2c, β1, β2,* and *β3*), and TH was evaluated by RT-PCR in fresh tissue as well as in isolated IVD cells. For RNA isolation, the tissue was minced with a scalpel and further processed according to manufacturers’ instructions (NucleoSpin^®^ RNA/Protein, Macherey and Nagel, Düren, North Rhine-Westphalia, Germany). Isolated IVD cells did not require special pre-treatments before RNA isolation. cDNA synthesis was performed using Quantabio qScript cDNA Synthesis Kit (VWR, Darmstadt, Hessen, Germany), and about 5 ng of cDNA per optimized primer were used for PCR (Taq PCR Master Mix, Qiagen, Hilden, North Rhine-Westphalia, Germany). All primers were obtained from Thermo Fisher Scientific (Darmstadt, Hessen, Germany, Table 3). The results were visualized by agarose gel electrophoresis with GelRed (Biotium, Fremont, California, USA) and ChemiDoc XRS+ (BioRad, Dreieich, Hessen, Germany). Human *GAPDH* served as a housekeeping gene. The PCR gels were analyzed densitometrically based on the intensities of corresponding PCR bands using the Image Lab software (BioRad, Dreieich, Hessen, Germany). In step 1, *GAPDH* band intensity of each individual patient was defined as “1” and AR expression levels were calculated in relation to that value. Then, semiquantitative relative gene expression levels depending on the grade of degeneration were calculated using grade I samples as a calibrator (mean of grade I results was defined as “1”), and correlations were performed with the degree of degeneration. Genes that did not show a detectable Ct value after 40 amplification cycles were deemed to have “zero “expression.

### 4.4. Immunohistological Stainings

The protein expression and localization of 3 major adrenoceptors (α2a-AR and β2-AR) in human and murine IVDs was investigated immunohistochemicaly. Human IVD-tissue samples were fixed in 4% paraformaldehyde (Merck, Darmstadt, Hessen, Germany) in 1x PBS overnight and after rinsed 3 times for 10 min with ddH_2_O embedded in paraffin (Paraplast PLUS, Merck, Darmstadt, Hessen, Germany). Sections of 8 μm were then dewaxed using a standard protocol with xylene and gradually rehydrated. After antigen demasking using citrate buffer (10 mM sodium citrate, 0.05% tween 20, pH 6 (Merck, Darmstadt, Hessen, Germany)) for 20 min at 95 °C, the endogenous peroxidase (0.3% H2O2 10 min, room temperature (Merck, Darmstadt, Hessen, Germany) enzyme was blocked as well as non-specific binding sites using secondary antibody-specific horse serum (VECTASTAIN® ABC-AP Staining KIT, Vector Labs or HRP-AEC Kit, Linaris, Dossenheim, Baden-Wurttemberg, Germany) for 45 min at room temperature. Sections were then incubated with the primary antibodies rabbit anti α2a-AR (1:200; ab85570), rabbit anti α2c-AR (1:200; ab151618; Abcam, Cambridge, United Kingdom), rabbit anti β2-AR (1:200; ab213651; Abcam, Cambridge, United Kingdom), at 4 °C overnight. Specific staining was visualized using horseradish peroxidase labeled secondary antibodies and peroxidase substrate solution (Vector Labs or HRP-AEC Kit, Linaris, Dossenheim, Baden-Wurttemberg, Germany). Control experiments were performed with unspecific isotype antibodies. AR-positive cells were counted in all stained sections, and different IVD areas (annulus vs. nucleus), as well as the two mouse genotypes, were compared regarding the level of AR expression. Additional sections were stained with dimethyl methylene blue (DMMB) or safranin-O/fast green to visualize tissue structure and proteoglycan distribution.

Furthermore, the expression of ECM proteins was analyzed in human and murine IVDs (type II collagen—Col II, type XII collagen—Col XII, cartilage oligomeric matrix protein—COMP, decorin—DCN). In the case of the Col II staining, the sections were digested using pepsin (0.025% in 0.2M HCL; Merck, Darmstadt, Hessen, Germany) for 15 min at 37 °C at first. An enzymatic digestion with hyaluronidase (500U/mL in Hyaluronidase Puffer pH 5.0; Merck, Darmstadt, Hessen, Germany) for 30 min at 37 °C and Proteinase K (10µg/mL in Proteinase Puffer pH 7.4, Thermo Fisher Scientific, Darmstadt, Hessen, Germany) for 10 min at 55 °C was conducted for all the antibodies to demask the antigens. After quenching the endogenous peroxidase with 3% H_2_O_2_ for 10 min and blocking with a blocking solution (Zytomed blocking solution, Zytomed systems ZUC025-100, Berlin, Germany) for 5 min at room temperature, the sections were incubated with the primary antibodies mouse anti-Col II (1:500; CP18, Merck, Darmstadt, Hessen, Germany), rabbit anti-Col XII (1:3000; [45], rabbit anti-COMP4-1 (1:500; Immundiagonstik AG, Bensheim, Hessen, Germany [46]), rabbit anti-decorin (1:500; [47]) at 4 °C over night. HRP-Polymer anti-rabbit or anti-mouse secondary antibodies (ZytoChem Plus, Berlin, Germany) as well as 3,3′-diaminobenzidine (0.05% DAB and 0.015% H_2_O_2_ in 0,01M PBS pH 7.2; Merck, Darmstadt, Hessen, Germany) was used to detect the specific primary antibody bindings. The brownish staining through the oxidation of the DAB was complemented with a nuclear counterstaining with Mayer’s Heamatoxylin (Merck, Darmstadt, Hessen, Germany) for 10 s at room temperature.

Murine samples were processed and stained the same way as the human tissue.

### 4.5. β2-AR Western Blot

IVD tissue and cell protein samples were loaded onto 10% SDS-PAGE and electro-transferred to a polyvinylidene difluoride (PVDF) membrane. Membranes were blocked with 5% bovine serum albumin for 1 h at room temperature before incubation with primary antibody for β2-AR (ab213651; Abcam, Cambridge, United Kingdom) at 4 °C overnight. The membranes were washed with TBST (Tris-buffered saline with Tween20) and incubated with HRP-conjugated secondary antibody (Dako Denmark A/S, Glostrup, Denmark) for 1 h at room temperature. The target protein expression was detected using the chemiluminescence (Clarity Western ECL Substrate, BioRad, Dreieich, Hessen, Germany) reagent. A cell lysate of the human squamous carcinoma cell line A431 (Abcam, Cambridge, United Kingdom) was used as a positive control (see also https://www.abcam.com/beta-2-adrenergic-receptor-antibody-epr707n-ab182136.html as additional information).

### 4.6. IVD Cell Isolation and Stimulation with NE

According to a method by Tang et al. [48], the IVD tissue was minced into 2 to 3 mm pieces with a sterile scalpel and placed in 75 cm^2^ cell culture flask and cultivated in Dulbecco’s modified Eagle medium (DMEM/F12; Gibco, Thermo Fisher Scientific, Darmstadt, Hessen, Germany) [41] with 1% penicillin/streptomycin (P/S) (Gibco, Thermo Fisher Scientific, Darmstadt, Hessen, Germany) and 10% FBS (Gibco, Thermo Fisher Scientific, Darmstadt, Hessen, Germany) at 37 °C in a humidified atmosphere containing 5% CO_2_ and 2% O_2_. After achieving confluence, cells were detached, and pellets were stored at −80 °C until PCR analysis. The used intervertebral discs were moderately degenerated (grade II).

In order to examine whether isolated IVD cells responded to NE, the 2 major AR-dependent signaling pathways, the phosphorylation of PKA and ERK1/2, were investigated. Cells were treated for 5 and 15 min with NE (10^−8^ or 10^−6^ M, Sigma-Aldrich, St. Louis, Missouri, USA). Protein isolation was performed using NucleoSpin RNA/Protein kit (Macherey Nagel, Düren, North Rhine-Westphalia, Germany).

### 4.7. NE-Dependent Signal Transduction

IVD protein samples were loaded onto 10% SDS-Page and electro-transferred to a polyvinylidene difluoride (PVDF) membrane. Membranes were blocked with 5% bovine serum albumin for 1 h at room temperature before incubation with primary antibodies for total ERK (#9170; Cell Signaling Technology), phosphorylated ERK (#4370; Cell Signaling Technology, Danvers, Massachusetts, USA), total PKA (#32514; Abcam, Cambridge, United Kingdom), phosphorylated PKA (#32390; Abcam, Cambridge, United Kingdom), and GAPDH (#(MA5-15738); Thermo Fisher Scientific, Darmstadt, Hessen, Germany) at 4 °C overnight. The membranes were washed with TBST and incubated with HRP-conjugated secondary antibody (Dako Denmark A/S, Glostrup, Denmark) for 1 h at room temperature. The target protein expression was detected using the chemiluminescence (Clarity Western ECL Substrate, BioRad, Dreieich, Hessen, Germany) reagent, with GAPDH as the endogenous control.

### 4.8. Statistical Analysis

All quantitative gene expression experiments were carried out with samples of at least 5 patients. Data were presented as box plots with medians. Multiple comparisons between groups of different degrees of degeneration were carried out using ANOVA on ranks or Wilcoxon/Mann–Whitney-Test followed by Bonferroni or Dunn’s correction. To analyze associations between AR expression and grade of degeneration, Spearman rank order correlations were performed. p-values less than 0.05 were considered significant. All statistical analyses were performed using SigmaPlot 14.0 software (Systat Software GmbH, Erkrath, Germany).

## Figures and Tables

**Table 1:** AR gene expression distribution in human IVD tissues with different degrees of degeneration (*n* = 43).

AR Subtype	Total (*n* = 43)		Grade I (*n* = 5)		Grade II (*n* = 9)		Grade III (*n* = 21)		Grade IV (*n* = 8)	
	*n*	%	*n*	%	*n*	%	*n*	%	*n*	%
*α1a*	42	97.7%	5	100%	8	88.9%	21	100%	8	100%
*α1b*	20	46.5%	2	40%	5	55.6%	8	38.1%	5	62.5%
*α2a*	39	90.7%	4	80%	7	77.8%	21	100%	7	87.5%
*α2b*	42	97.7%	4	80%	9	100%	21	100%	8	100%
*α2c*	42	97.7%	5	100%	9	100%	20	95.2%	8	100%
*β1*	43	100%	5	100%	9	100%	21	100%	8	100%
*β2*	43	100%	5	100%	9	100%	21	100%	8	100%

**Table 2:** Characteristics of patients under study.

Patient Characteristics	Number (%)/Mean Age ± SEM
total (number/age)	43 (100%)/66.93 ± 1.83
female (number/age)	33 (76.74%)/66.64 ± 2.21
male (number/age)	10 (23.26%)/67.9 ± 2.9

**Table 3:** The primers used for PCR

Gene Symbol	NCBI Reference	Foward (5′−3’)	Reverse (5´−3´)
*GAPDH*	NM_001289745.2	CTCCTGTTCGACAGTCAGCC	TTCCCGTTCTCAGCCTTGAC
*ADRA1A*	NM_000680.3	CCATGCTCCAGCCAAGAGTT	TCCTGTCCTAGACTTCCTCCC
*ADRA1B*	NM_000679.3	GTCCACCGTCATCTCCATCG	GAACAAGGAGCCAAGCGGTAG
*ADRA1D*	NM_000678.3	TGACTTTCCGCGATCTCCTG	TTACCTGCCACGGCCATAAG
*ADRA2A*	NM_000681.3	TGGTCATCGGAGTGTTCGTG	GCCCACTAGGAAGATGGCTC
*ADRA2B*	NM_000682.6	GACATTTCACCGGCAACACC	GGGACTGAGAACCAGGAAGC
*ADRA2C*	NM000683.3	CGATGTGCTGTTTTGCACCT	GGATGTACCAGGTCTCGTCG
*ADRB1*	NM_000684.2	TAGCAGGTGAACTCGAAGCC	ATCTTCCACTCCGGTCCTCT
*ADRB2*	NM_000024.5	CAGAGCCTGCTGACCAAGAA	GCCTAACGTCTTGAGGGCTT
*ADRB3*	NM_000025.2	GCCAATTCTGCCTTCAACCC	GCCAGAGGTTTTCCACAGGT
*TH*	NM_000360.3	CAGGCAGAGGCCATCATGT	GTGGTCCAAGTCCAGGTCAG

## Supplementary Materials

Supplementary Materials can be found at https://www.mdpi.com/1422-0067/21/6/2085/s1.

## Abbreviations

AF	Annulus fibrosus
AR	Adrenoceptor
cDNA	Complementary desoxy ribonucleic acid
Col II	Type II collagen
Col VI	Type VI collagen
Col XII	Type XII collagen
COMP	Cartilage oligomeric matrix protein
DCN	Decorin
DMEM/F12	Dulbecco’s modified eagle’s medium and Ham’s F-12 Medium
DMMB	1,9-Dimethyl-methylene blue
ECM	Extracellular matrix
ERK	Extracellular signal-regulated kinases
FBS	Fetal bovine serum
GAPDH	Glyceraldehyde-3-phosphate dehydrogenase
HT29	Human colon cancer cell line
IL	Interleukin
IVD	Intervertebral disc
IVDD	Intervertebral disc degeneration
mRNA	Messenger ribonucleic acid
NE	Norepinephrine
NP	Nucleus pulposus
OA	Osteoarthritis
P/S	Penicillin streptomycin
PCR	Polymerase chain reaction
pERK	Phosphorylated extracellular signal-regulated kinases
PKA	Protein kinase A
pPKA	Phosphorylated protein kinase A
RT-PCR	Reverse-transcriptase PCR
Saf-O	Safranin-O/fast green staining
sGAG	Sulphated glycosaminoglycans
TH	Tyrosine hydroxylase
WT	Wildtype mice
