# Supplementary material for: Adrenoceptor Expression during Intervertebral Disc Degeneration
Source: Int J Mol Sci. 2020 Mar 18;21(6):2085. doi: 10.3390/ijms21062085 (PMC7139977; doi:10.3390/ijms21062085)
Supplement: Supplementary file 1 [file ijms-21-02085-s001.pdf]

Supplementary Material - ijms-707703

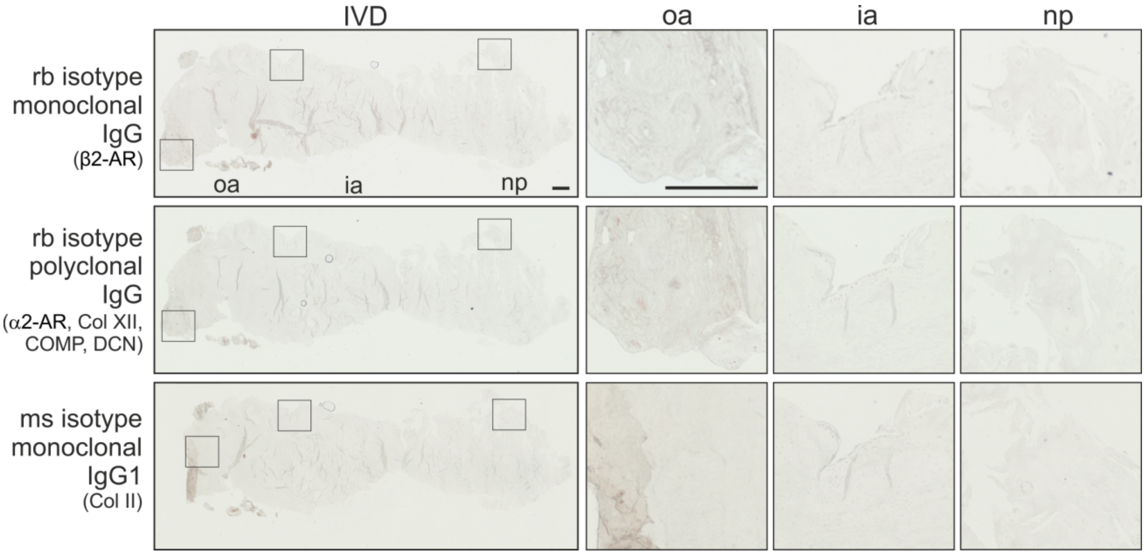

Supplementary figure 1. Isotype controls for the respective immunohistochemical staining in human IVD sections (bars 500 µm). Squares in the left panel indicate further magnified regions shown to the right. Abbreviations: oa – outer annulus, ia – inner annulus, np – nucleus pulposus, Col II - type II collagen, Col XII - type XII collagen, COMP - cartilage oligomeric matrix protein, DCN - decorin

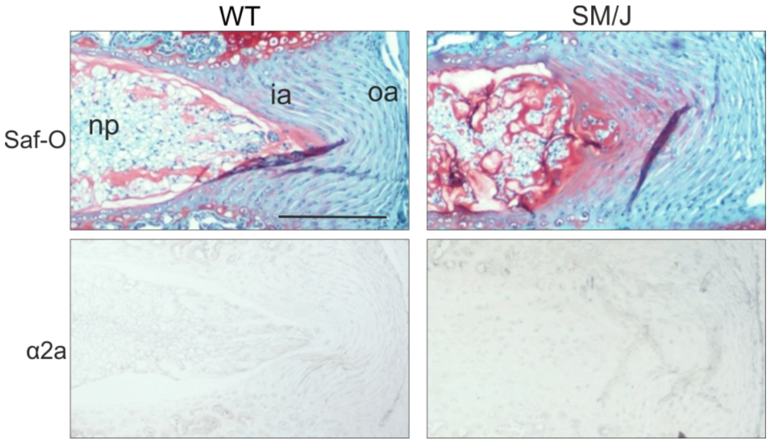

Supplementary figure 2. Immunohistochemical analysis of α2a-AR in IVD sections of WT and SM/J mice (bars 500 µm). Abbreviations: Saf-O – safranin-O staining, oa – outer annulus, ia – inner annulus, np – nucleus pulposus

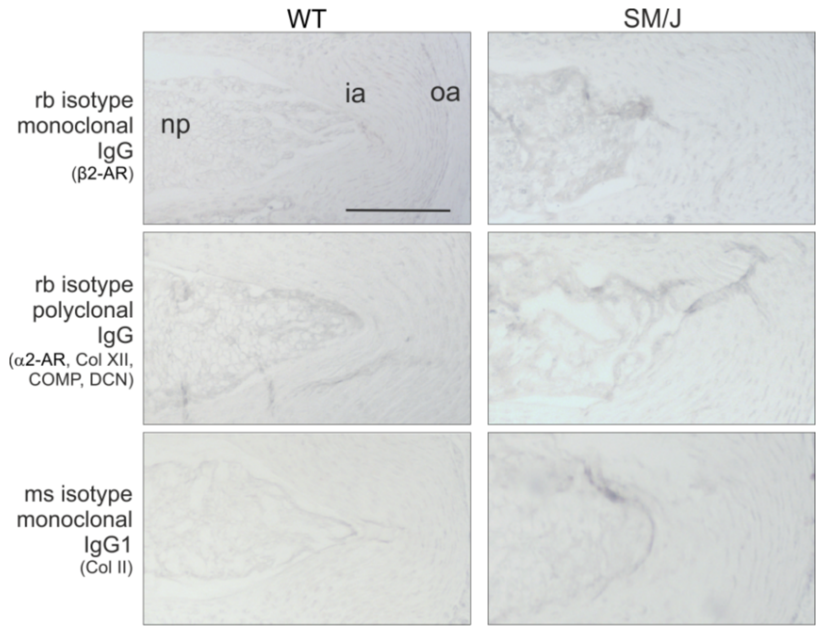

Supplementary figure 3. Isotype controls for the respective immunohistochemical staining in murine IVD sections (bars 500 µm). Col II - type II collagen, Col XII - type XII collagen, COMP - cartilage oligomeric matrix protein, DCN – decorin

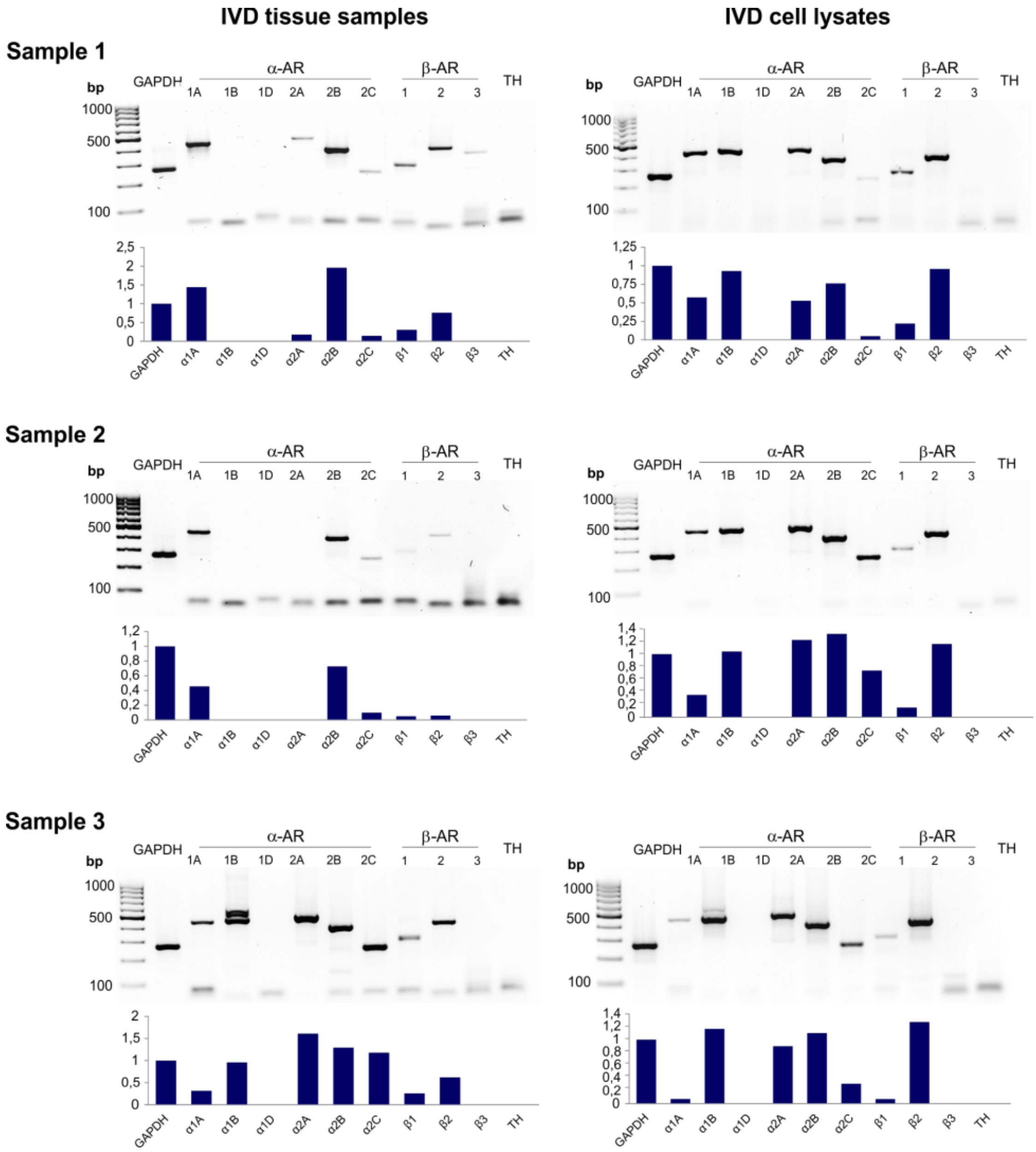

Supplementary figure 4. Adrenoceptor (AR) and tyrosine hydroxylase (TH) gene expression in human IVD tissue and in corresponding isolated IVD cells analyzed by RT-PCR from three samples. Gene expression of all known AR subtypes as well as of TH in human IVD tissue samples (representative pictures of human IVD samples; grade II of degeneration, (n=3)). GAPDH band intensity of each individual patient was defined as „1" and AR expression levels were calculated in relation to that value.

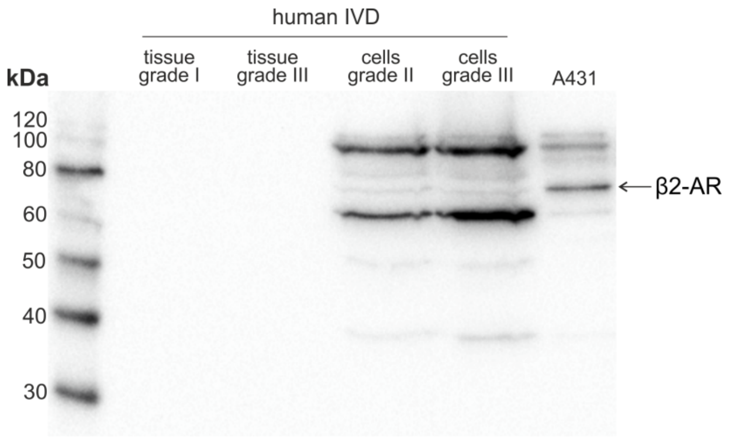

Supplementary figure 5. Protein expression of β2-AR examined by western blot analysis. Immunoblot of β2-AR protein in extracts from representative IVD tissue (grade I and III) and IVD cells (isolated from grade II tissues)(A431=positive control, see also https://www.abcam.com/beta-2-adrenergic-receptor-antibody-epr707n-ab182136.htm).
